# Investigation Into an Outbreak of Dengue-like Illness in Pernambuco, Brazil, Revealed a Cocirculation of Zika, Chikungunya, and Dengue Virus Type 1

**DOI:** 10.1097/MD.0000000000003201

**Published:** 2016-03-25

**Authors:** Rodrigo Pessôa, João Veras Patriota, Maria de Lourdes de Souza, Alvina Clara Felix, Nubia Mamede, Sabri S. Sanabani

**Affiliations:** From the Clinical Laboratory, Department of Pathology (RP, SSS), Hospital das Clínicas (HC), School of Medicine, University of São Paulo, São Paulo; Municipal Hospital of Tuparetama (JVP, MDLDS, ACF, NM), Pernambuco; and Department of Virology (SSS), São Paulo Institute of Tropical Medicine, São Paulo, Brazil.

## Abstract

In April 2015, an outbreak of dengue-like illness occurred in Tuparetama, a small city in the northeast region of Brazil; this outbreak was characterized by its fast expansion. An investigation was initiated to identify the viral etiologies and advise the health authorities on implementing control measures to contain the outbreak. This is the first report of this outbreak in the northeast, even though a few cases were documented earlier in a neighboring city.

Plasma samples were obtained from 77 suspected dengue patients attending the main hospital in the city. Laboratory assays, such as real-time reverse transcription polymerase chain reaction, virus cDNA sequencing, and enzyme-linked immunosorbent assay, were employed to identify the infecting virus and molecular phylogenetic analysis was performed to define the circulating viral genotypes.

RNA of Zika virus (ZIKV) and Dengue virus (DENV) or IgM antibodies (Abs) to DENV or chikungunya (CHIKV) were detected in 40 of the 77 plasma samples (51.9%). DENV was found in 9 patients (11.7%), ZIKV was found in 31 patients (40.2%), CHIKV in 1 patient (1.3%), and coinfection of DENV and ZIKV was detected in 2 patients (2.6%). The phylogenetic analysis of 2 available partial DENV and 14 ZIKV sequences revealed the identities of genotype 1 and the Asiatic lineage, respectively.

Consistent with recent reports from the same region, our results showed that the ongoing outbreak is caused by ZIKV, DENV, and CHIKV. This emphasizes the need for a routine and differential diagnosis of arboviruses in patients with dengue-like illness. Coordinated efforts are necessary to contain the outbreak. Continued surveillance will be important to assess the effectiveness of current and future prevention strategies.

## INTRODUCTION

Zika virus (ZIKV) is an emerging arthropod-borne virus (arbovirus) belonging to the family Flaviviridae and genus *Flavivirus*. The virus was first isolated from an infected rhesus monkey in the Zika forest of Uganda in 1947.^1^ Subsequently, sporadic isolations have been obtained from humans and mosquito species in both Africa and Asia.^2^ In 2007, ZIKV caused an outbreak of relatively mild disease characterized by rash, arthralgia, and conjunctivitis in Yap State, Federated States of Micronesia.^3^ Recently (October 2013), large outbreaks in New Caledonia (1400 confirmed cases), Cook Islands (>900 cases), and Easter Island have been reported.^4^ Infection with ZIKV has been reported among travelers returning from nonendemic countries, including Japan, Germany, Italy, Canada, Australia, and the United States^5–9^ and from endemic countries such as Brazil.^8^ Phylogenetic analysis of available sequences suggests that African and Asian strains have emerged as 2 distinct lineages.^2,7^ ZIKV is a mosquito-borne pathogen maintained in a sylvatic cycle comprising nonhuman primates and several mosquito vectors of the *Aedes* species (*A africanus*, *A aegypti*, and others).^10–12^ The fact that *A aegypti* is a permissive vector of dengue virus makes it conceivable that ZIKV virus can also circulate in areas where dengue is endemic. ZIKV transmission has been reported to occur through sexual contact^13^ and perinatal transmission.^14^ Symptoms of ZIKV may include fever, maculopapular rash, headache, malaise, fatigue or myalgia, and arthritis and arthralgia.^15^ These symptoms can easily be confused with those of dengue infection and might be misdiagnosed during local dengue outbreaks, thus leading to underreported prevalence of ZIKV-associated illness.^5,13^ The illness is usually mild and lasts 4 to 7 days.

Like all flaviviruses, ZIKV has a positive-sense, single-stranded RNA genome of 10,794 bases in length.^16^ The genome contains a single open reading frame (ORF) that encodes a polyprotein with 3 structural proteins (the capsid [C], premembrane/membrane [prM], and envelope [E]) and 7 nonstructural proteins (NS1, NS2A, NS2B, NS3, NS4A, 2K, NS4B, and NS5).^16^

Dengue, a mosquito-borne disease caused by a flavivirus, is now prevalent in over 100 countries in Africa, the America, the Eastern Mediterranean, Southeast Asia, and the Western Pacific. The dramatic increase of the incidence and geographic expansion of dengue around the world is posing a threat to approximately 2.5 billion people in dengue-endemic countries.^17^ The infected population is about 390 million cases per year (95% credible interval: 284–528 million), of which 96 million (67–136 million) manifest clinically (with any severity of disease).^18^ Dengue viruses (DENVs) are small, single-stranded RNA viruses belong to the family Flaviridae and display 5 distinct serotypes (DENV-1 to -5), all of which can classically cause undifferentiated fever.^19,20^ Current epidemiological data indicate that DENV-2 is the most relevant serotype worldwide because of its connections with the largest outbreaks; it is followed in sequence by DENV-3, DENV-1, and DENV-4.^21^ In Brazil, since the first confirmation of a DENV epidemic in Boa Vista, state of Roraima, Amazon region, in 1981, dengue outbreaks have been reported in practically all regions of the country.^22,23^ Recent data (2014) from the World Health Organization (WHO) indicated that Brazil had the highest incidence rate of dengue (294.02/100,000 inhabitants) among the countries of the Southern Cone (Argentina, Brazil, Chile, Paraguay, and Uruguay) (Pan American Health Organization/WHO, Dengue Cases, Americas, 2014, http://www.paho.org/). In addition to DENV, the Brazilian government has reported 3 imported cases of chikungunya virus in 2010. A cocirculation of ZIKV in association with an ongoing outbreak of an acute maculoexantematic illness in the northeast region of Brazil has recently been reported.^24,25^ In October 2015, the Brazil Ministry of Health (BMH) reported an unusual increase in cases of microcephaly in the northeastern state of Pernambuco and lower levels in other northeastern states. On 17 November, BMH confirmed the presence of ZIKV (using reverse transcription polymerase chain reaction [RT-PCR] nn g ng) in amniotic fluid samples collected from 2 fetuses with microcephaly in the northeast. Both pregnant women presented symptoms of ZIKV infection during early pregnancy. Possible associations between ZIKV infection in pregnancy and fetal microcephaly are under investigation. The current data from BMH confirmed the circulation of ZIKV in 18 of the 26 Brazilian states.

In May 2015, our laboratory was notified by colleagues from the secretary of health in the city of Tuparetama in the northeast region of Brazil of unusual increase in new cases of maculopapular rash, conjunctivitis, joint pain, and other symptoms that include mild fever lasting for 2 to 7 days. After initiating this investigation, other studies were conducted using samples from a similar outbreak in the city of Camaçari and Salvador, state of Bahia in northeast of Brazil. The results of these studies were recently published and have confirmed a c-circulation of ZIKV, CHIKV, and DENV.^24,25^

## MATERIALS AND METHODS

### Ethical Approval

Study methods were ethically reviewed and approved by the São Paulo University Faculty of Medicine Review Board (FMUSP N0 1.184.947). All study participants provided informed consent. The aims of our study were explained, and written informed consent was obtained from all participants. Parents or legal guardians provided written informed consent on behalf of the children.

### Setting

**FIGURE 1:**
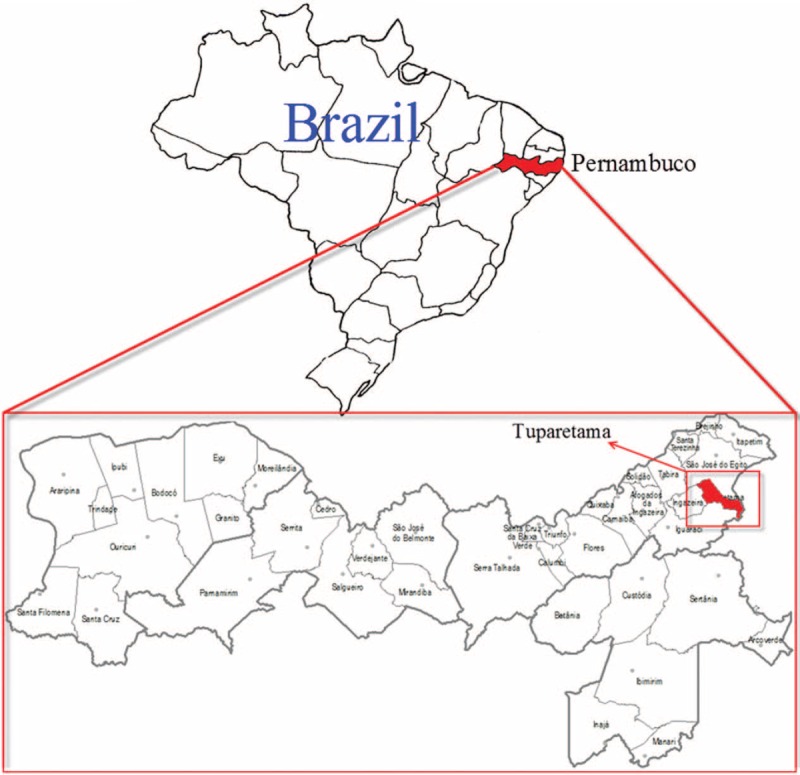
Map of Brazil showing the geographical location of Tuparetama city (red colored) and Pernambuco State (red colored), where samples of the current outbreak were collected during May 25, 2015 to May 31, 2015.

The city of Tuparetama (07° 36’ 08“ South, 37°18’ 41” West) is a small municipality in the state of Pernambuco, northeast of Brazil, with an area of 186 km^2^ according to the Brazilian Institute of Geography and Statistics (Figure 1). The municipality had approximately 8678 inhabitants during the last census of 2009, with population density of 44.4 inhabitants per square kilometer. Like other municipalities in northeast of Brazil, the rainy season in Tuparetama coincides with the summer period, mostly in January through March, when temperatures range from 38 °C to 45 °C. The housing is mostly low-rise, self-constructed buildings that are regularly distributed. The residents have a habit of cultivating water plants and storing water in their homes for plant watering. The hygiene situation is generally good.

### Study Participants and Procedures

Consecutive adult patients presenting to the outpatient department of Severino de Souto Siqueira Hospital from May 25 to 31, 2015 were recruited to the study. Patients with rash, conjunctivitis, and/or joint pain with or without fever or headache were examined further. Clinical diagnosis and patient management were performed according to the local standard of care and were recorded on a standard assessment form. Symptomatic dengue was defined as acute febrile illness plus a positive IgM or RT-PCR with a follow-up positive IgG test in the serosurvey. Inapparent dengue infection was defined as having a positive dengue test (IgM, or RT-PCR) but no febrile illness. Confirmed cases of CHIKV infection were defined based on considered reactivity to IgM enzyme-linked immunosorbent assay (ELISA) and the presence of viral RNA in clinical samples. Patients whose specimens tested positive for anti-chikungunya IgM only by ELISA were designated as probable cases. Ten milliliters of intravenous blood samples were obtained from 77 eligible patients. These samples were collected aseptically into tubes with ethylenediaminetetraacetic acid. All samples were temporarily kept at −20 °C at the local hospital before being transported to our laboratory at the São Paulo Institute of Tropical Medicine, where they were stored at −80 °C until analysis.

### Laboratory Diagnosis and Virus Identification

#### Detection of DENV Abs (IgM and IgG) and CHIKV Abs (IgM)

Anti-dengue virus IgM Abs in plasma samples were detected by an IgM-capture ELISA kit (dengue virus IgM capture DxSelect; Focus Diagnosis, CA) according to the manufacturer's protocol. Anti-dengue virus IgG Abs were detected by ELISA using an IgG indirect ELISA kit (Dengue IgG indirect ELISA; Panbio Ltd, Queensland, Australia) according to the manufacturer's protocol. The assays for detection of anti-dengue virus IgG Abs were used only in the follow-up serosurvey of patients who had detectable anti-dengue virus IgM Abs. All plasma samples were also screened for CHIKV IgM Abs using IgM IIFT from EUROIMMUN AG. Plasma was considered positive only when the optical density exceeded 1.1 times that of the negative control.

#### Extraction of Viral RNA and cDNA Synthesis

Total RNA was extracted from 140 μL serum samples by using the QIAamp viral RNA kit (Qiagen, Hilden, Germany) according to the manufacturer's instructions. The RNA was extracted in 60 μL of elution buffer and was used immediately to synthesize the cDNA with the Superscript III (Invitrogen, Carlsbad, CA) according to the manufacturer's protocol. Briefly, a master mix consisting of 0.1 mmol/L deoxynucleoside triphosphates, 150 ng of random hexamers, 5 μL of 10 × buffer, 10 mmol/L dithiothreitol, 2.5 U of RNaseOUT recombinant RNase inhibitor (Promega, Madison, WI), and 5 U of Superscript III (Invitrogen, Carlsbad, CA) reverse transcriptase enzyme was prepared. A 10-μL aliquot of extracted RNA was added to this mixture, and the reaction was incubated at 25°C for 5 minutes, 42 °C for 50minutes, and then 95°C for 3 minutes. The resultant cDNA was stored at −20°C for future use.

#### Real-time RT-PCR for DENV

Dengue RNA genomes from all 4 serotypes were examined by a previously published real-time PCR method.^26^ Briefly, A total of 7.5 μL of extracted RNA was used as a template in a 15-μL reaction volume using SuperScript III Platinum SYBR green 1-step quantitative RT-PCR (qRT-PCR) with the ROX Kit (Applied Biosystems, Austin, TX) and 0.4 μmol/L of pan-dengue primers.^27^ The samples were amplified in an ABI Prism 7300 sequence detection system (PE Applied Biosystems, Foster City, CA). The qRT-PCR assay consisted of a 10-minute reverse transcription step at 60°C and then 1 minute for *Taq* polymerase activation at 95 °C, followed by 45 cycles of PCR at 95°C without holding time, 60°C for 3 s, and 72°C for 10 s.

#### Real-time PCR for Detection of Other Flaviviruses

The viral genomes of yellow fever virus (YFV), Japanese encephalitis virus (JEV), West Nile virus (WNV), St Louis encephalitis virus (SLEV), and dengue virus (DENV) serotypes 1 to 4 in serum samples were investigated by a previously described multiplex real-time RT-PCR with SYBR green I dye instead of probes.^28^ All transcribed viral RNA was used as a template to amplify a 266-base-pair (bp) fragment together with positive (dengue 3) and negative (nuclease-free water) controls included in each assay. Due to the lack of positive controls for YFV, WNV, JEV, and SLEV, we were unfortunately not able to differentiate the viral species by melting analysis. We thus decided to sequence any amplified products of the expected size. To this end, amplified fragments were purified using a QIAquick PCR Purification Kit (Qiagen, Hilden, Germany). The purified products were directly sequenced and submitted for phylogenetic analysis to identify flavivirus type.

#### Real-Time PCR for Detection of ZIKV and CHIKV

The primary analysis of the nucleotide sequences generated by the multiplex PCR of flavivirus revealed the presence of ZIKV, which was found to be highly homologous to the Asian lineage of (GenBank no. KF993678). These results allowed us to design a specific sybr green qRT-PCR assay for the ZIKV. The forward ZE_FF_8912 5’ GAA GCC CTT GGA TTC TTG AAC GAG G’3 and reverse primers ZE_RR_9024 5’ CGA CTC ATC TCT TCT AGG ACA TAT CC’3 were designed using Oligo 6.0 software (Molecular Biology Insights, Cascade, CO) and were targeted to the NS5 region. The standard curve was generated by a 10-fold dilution of a PCR product of 737 bp from a ZIKV-positive sample in the range of 1 to 10^5^ copies/μL. The forward and reverse primers use to generate this fragment are ZF (RTCURVE) 8864 5’ GCC GCG CCA TCT GGT ATA TGT GG’3 and ZSEQR_9601 5’GCT TGT TGA AGT GGT GGG AGC’ 3. The amplifications were performed based on the SYBR technique by using the KAPA SYBR FAST qPCR Kit 2X Master MIX (Kapa Biosystems Inc., Woburn, MA) in an ABI Prism 7300 sequence detection system (PE Applied Biosystems). Reactions were undertaken with an initial 5 minutes at 95°C, followed by 40 cycles of denaturation at 95°C for 30 s, annealing at 60°C for 45 s. The plasma samples were also tested by qRT-PCR for the presence of CHIKV using previously published primers targeting the *nsp1* gene.^29^ The amplification conditions and reagents were the same as those used in the assay designed to detect ZIKV.

For all of our qRT-PCR assays, samples were considered positive when all 3 replicates had a quantification cycle (Cq) of ≤40 cycles and had a shape of the melting temperature identical to control. A sample with a single or duplicate positive result of a triplicate was repeated to confirm positivity; if all triplicates are negative/undetectable, the tests were reported as undetectable. Results were expressed as the mean Cq of the 3 replicates.

#### PCR Analyses for Amplification of DNA Fragment of ZIKV and Sequencing

To allow a more advanced phylogenetic analysis, an additional reverse primer ZRO_NFLG_10121 CGA ACT TGG GTG GAT AGG TAG TCC was designed to amplify a larger PCR fragment (689 bp). This primer was used in combination with the forward ZF (RTCURVE) 8864 in the first round of PCR. The PCR condition consisted of an initial denaturation step at 95°C for 3 minutes, followed by 35 cycles of 95°C for 20 s, 60°C for 20 s, and 68°C for 1 minute, followed by a final extension at 68°C for 7 minutes. For the nested PCR, 5 μL of the first PCR reaction was used; the PCR mix included ZEFF_8912 and ZESQR_9601 primers and the PCR conditions for the nested PCR reaction were identical to the first round.

### Sequencing and Phylogenetic Analysis

The amplified DNA fragments were purified using a QIAquick PCR Purification Kit (Qiagen, Hilden, Germany) and directly sequenced using the nested primers and the PRISM Big Dye Terminator Cycle Sequencing Ready Reaction Kit (Applied Biosystems/Perkin-Elmer, Foster City, CA) on an automated sequencer (ABI 3130, Applied Biosystems). After excluding the primer regions, each amplicon was assembled into a contiguous sequence alignment and edited with Sequencher 4.7 (Gene Code Corp, Ann Arbor, MI). The alignment of multiple sequences, including all publicly available ZIKV sequences deposited in Genbank, was performed using CLUSTAL X^30^ and followed by manual editing using BioEdit Sequence Alignment Editor version 5.0.7.^31^ Gaps and ambiguous positions were removed from the final alignment. The TN93 + G + I model was selected as the best-fitted model using MEGA 5.05^32^ and a maximum likelihood (ML) tree constructed using the PHYML v.3.0 algorithm^33^ with a BIONJ starting tree. Heuristic tree searches under the ML optimality criterion were performed using the NNI branch-swapping algorithm. The approximate likelihood ratio test (aLRT) based on a Shimodaira-Hasegawa-like procedure was used as a statistical test to calculate branch support. The phylogenetic tree was displayed using MEGA version 6.0.

### GenBank Accession Numbers

All consensus genome assemblies generated in this study were submitted to NCBI's GenBank database (Accession numbers KU232286-KU232301).

## RESULTS

The total number of patients enrolled in this study was 77, including 52 (67.5%) females and 25 (32.5%) males. Patients ranged in age from 1 to 80 years; 10 were younger than 10 years (13%, children), 12 were 10 to 19 years’ old (15.6%, adolescents), and 55 were older than 19 years (71.4%, adults). As shown in Table 1, the main symptoms reported for these patients were rash (77.9%), fever (68.8%), headache (66.2%), joint pain (64.9%), and conjunctivitis (61%). All these symptoms were reported by 18.2 % of the patients.

**TABLE 1:**
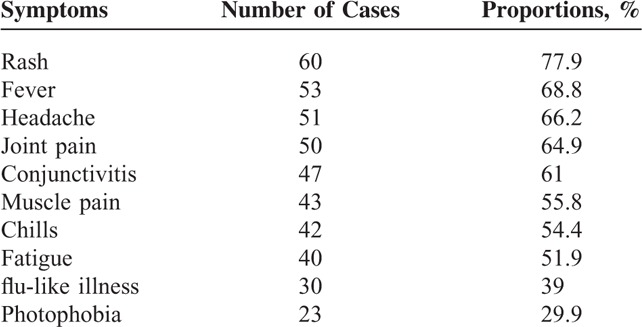
Symptom Composition of 77 Cases of the Investigated Patients in Tuparetama City

There were 9 (11.7%) laboratory-confirmed DENV cases. Two (2.6%) were confirmed with qRT-PCR, the other 7 (9.1%) were confirmed with IgM antibody detection in plasma, and 77.8% of these cases had fever and joint pain. Female patients comprised 55.5% of these cases. The youngest patient was 8 years’ old and the oldest was 49 years’ old; the median age was 36 years. About 77.8% of the laboratory-confirmed DENV cases were in the adult age group (ranging 34–49 years’ old). All the 7 cases tested positive for DENV IgM Abs showed high IgG titers in follow-up specimens. Only 1 patient (059DENV_PEBR15) had a diagnosis of inapparent dengue infection (Table 2).

**TABLE 2:**
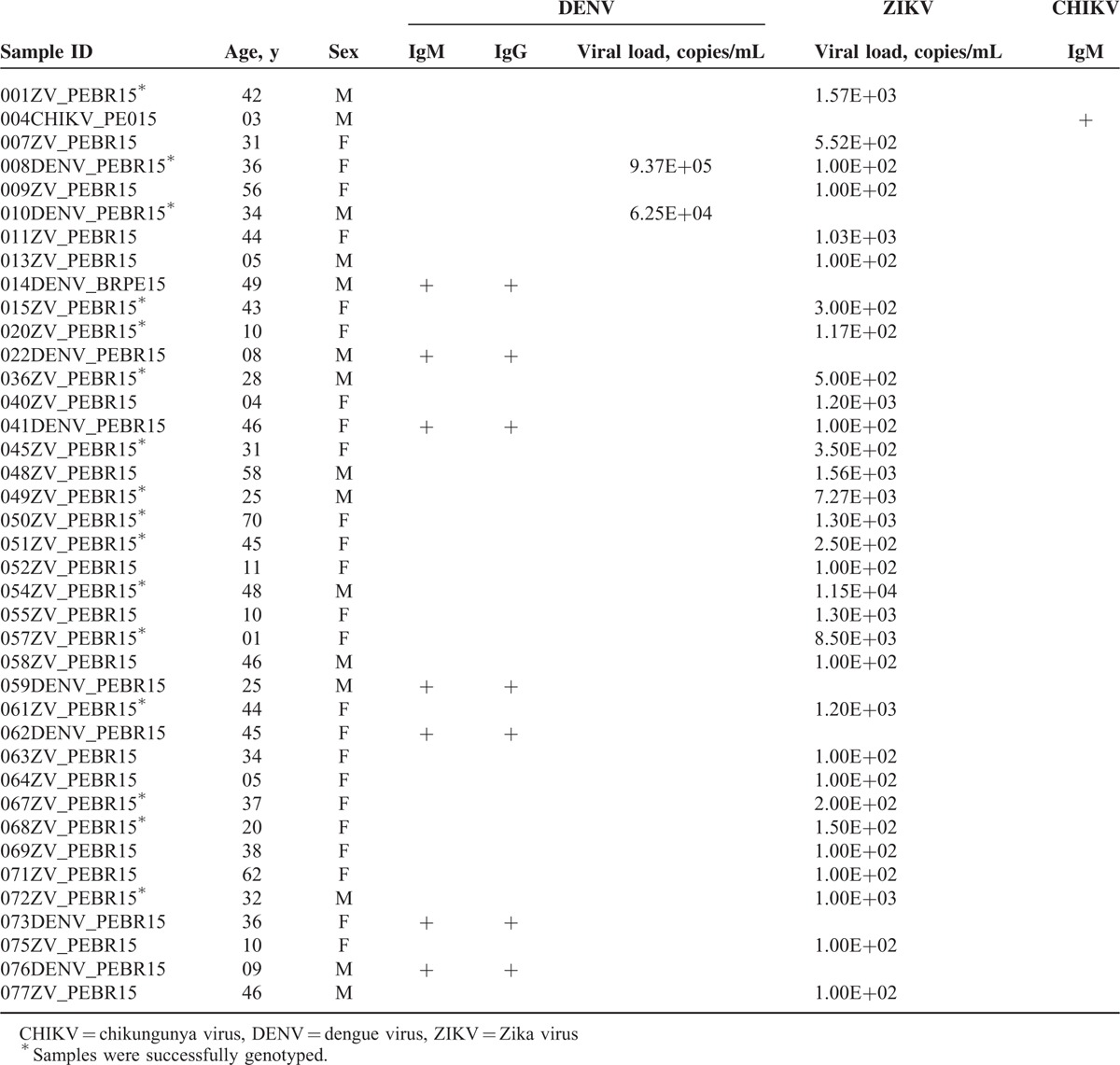
Characteristics of Patients Tested Positive for DENV, ZIKV, or CHIKV

In 31 of the 77 cases (40.2%), ZIKV diagnosis was confirmed by qRT-PCR. Of these patients, 83.9% had rash, 64.5% fever, 64.5% joint pain, and 54.8% had rash and fever. The virus was detected in 81% and 80% in the first 3 days after rash and fever onset, respectively. Females comprised the majority of patients, 71% of the cases. The youngest patient was 1-year old and the oldest was 70 years’ old, and the median age was 37 years. About 74.2% of the laboratory-confirmed ZIKV cases were in the adult age group (ranging 20–70 years’ old). The quantification of ZIKV RNA in clinical samples varied from 100 to 8.5 × 10^3^ copies/mL (Table 2).

Two samples (008ZV_PEBR15 and 041ZV_PEBR15) that were confirmed for DENV were also positive for ZIKV. Patient 008ZV_PEBR15 was positive for DENV and ZIKV by qRT-PCR, whereas patient 041ZV_PEBR15 was IgM-positive for DENV and had detectable ZIKV genomes. Our results thus confirm a coinfection of both viruses at a rate of 2.6%. All patients were negative by qRT-PCR for CHIKV1–4, YFV, JEV, WNV, and SLEV.

Only 1 patient (004CHIKV_PEBR15) tested positive for CHIKV IgM Abs when the qRT-PCR was negative. The patient had a rash and conjunctivitis, but no fever, at the time of sample collection.

**FIGURE 2:**
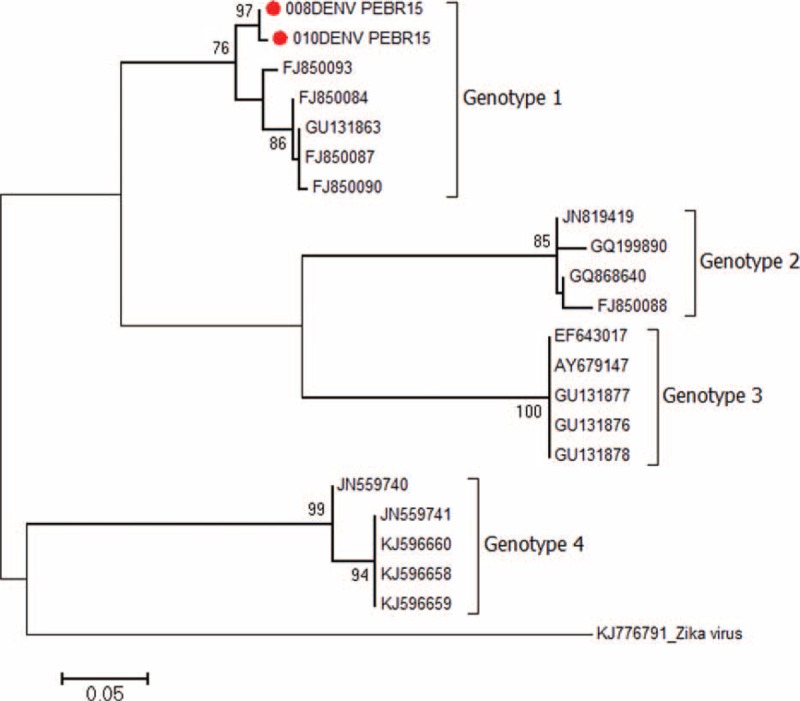
Maximum-likelihood phylogenetic tree of the 2 established dengue virus genotype 1 (DENV-1) sequences identified in this study (indicated by red circles). The tree is based on 266-nt partial NS5 nucleotide sequences. The tree was generated by the ML method using the TN93 + G + I model implemented in the PHYML v.3.0 package. The approximate likelihood ratio test (aLRT) values of at least 70% are indicated at nodes. The tree was rooted with the Zika virus (GenBank: KJ776791) lineage from French Polynesia. The scale bar represents 0.05 nucleotide substitutions per site.

Genotypes were determined from the 2 samples confirmed for DENV and assigned based on the portion of the NS5 gene sequenced^28^; these sequences, together with 20 published sequences from GenBank, provided a total of 22 sequences available for analysis (Figure 2). All of the sequences segregated into 1 of the 4 dengue genotypes, indicating that the short fragments of the NS5 gene used in this study were adequate to allow genotype identification. As depicted in Figure 2, the 2 DENV sequences positioned with the genotype 1 reference group (aLRT statistical value of 76%) showed nucleotide distances of 5%.

**FIGURE 3:**
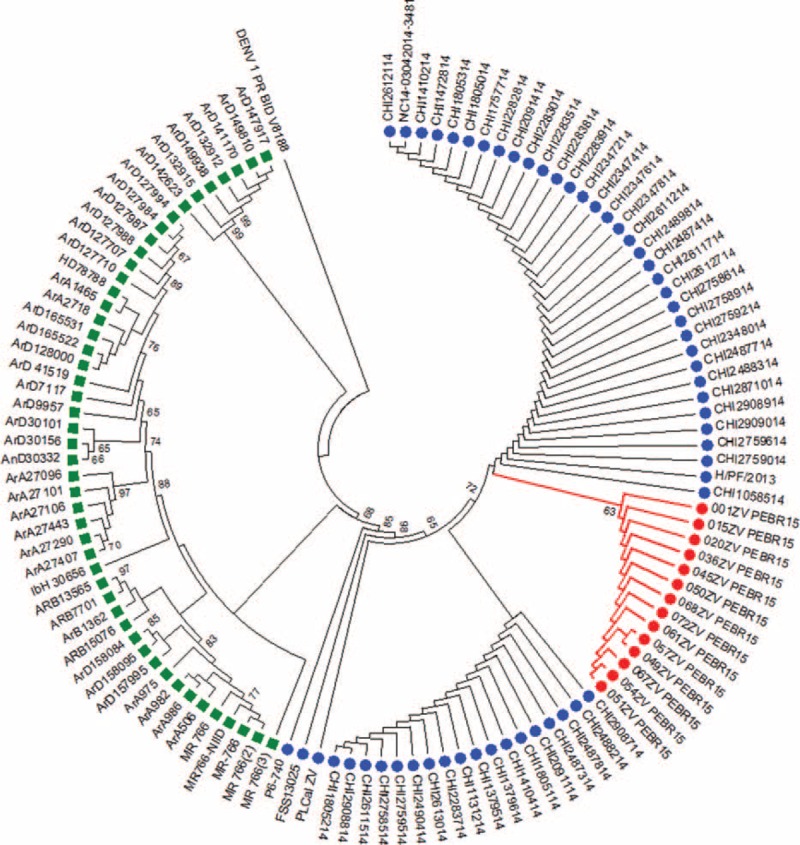
Phylogenetic tree constructed using a maximum-likelihood method from a partial NS5 region (689 bp; nt 8912–9601 of PLCal_ZV, GenBank: KF993678) of 14 samples from the present study (indicated by red circles), 51 sequences from Chile and 3 other Asiatic lineages (indicated by blue circle), and the 48 Zika virus sequences originated from Africa (indicated by green square). The tree was generated by the ML method using the TN93 + G + I model implemented in the PHYML v.3.0 package. The approximate likelihood ratio test (aLRT) values of at least 63% are indicated at nodes. The tree was rooted with the dengue virus (GenBank: KJ18936) genotype 1 from Puerto Rico.

Of the 31 plasma samples positive for ZIKV by qRT-PCR, the partial NS5 fragment from 14 samples was successfully genotyped. Sequence comparisons showed high degrees of identity among the 14 sequences; the paired identity at the nucleotide level ranged from 99.4% to 100%. When sequences were compared with ZIKV sequences, including recently published viral sequences from Chile^34^ and Asia,^35^ the identity values were 99.3% and 98.6% in the partial NS5 gene, respectively. When the sequences were compared with ZIKV sequences from West Africa, the nucleotide identities was 83.1%. The ML phylogenetic tree was then constructed using the 14 fragments sequenced in this study, the 51 sequences from Chile, and 51 other sequences from other parts of the world including West Africa. The ML tree depicted in Figure 3 shows the Brazilian ZIKV sequences formed a monophyletic cluster (63% aLRT) that groups with ZIKV from Chile (72% aLRT). This Latin American ZIKV cluster demonstrates strong clustering (86% aLRT) to the Asian lineages characterized in French Polynesia in 2013,^36^ as shown in Figure 3.

## DISCUSSION

This study demonstrates the cocirculation of DENV, ZIKV, and CHIKV and provides evidence of coinfection of DENV-ZIKV in the northeast region of Brazil. The high degree of similarity among the 14 partial sequences of the ZIKV NS5 gene suggests a single introduction of the virus into the human population. A few recent case reports and small studies implicated ZIKV with the outbreak of dengue-like illness currently ongoing in the northeast region of Brazil.^8,37^

Campos et al^24^ reported the association of ZIKV infection in 42% (10/24) of selected patients from Camaçari, northeast of Brazil, who had maculopapular rash, fever, myalgias, and headache. The same study also revealed cocirculation of CHIKV in 12.5% of the investigated cases. Another study on 54 serum samples from patients with acute exanthematous illness from Salvador, state of Bahia, showed simultaneous detection of ZIKV in 5.2% of the samples, CHIKV in 5.2% of the samples, DENV type 3 in 1.7% of the samples, and DENV type 4 in 1.7% of the samples. Recently, Guilherme et al^38^ reported the first autochthonous case of ZIKV infection in a HIV-infected patient in Rio de Janeiro, southeast of Brazil.

Our analysis demonstrated that ZIKV sequences obtained in this study are very close to the Chilean ZIKV strains with mean sequence divergence of 0.7%. These results may indicate that Brazilian ZIKV had its origin in Chile. The Chilean ZIKV strains were associated with the outbreak on Easter Island that began in January 2014.^34^ It is also likely that ZIKV could have spread from Southeast Asia to the Latin American countries because these isolates were closely related to the Asiatic clade in the phylogenetic tree. It is suspected that the virus was introduced during the soccer World Cup in 2014, although this hypothesis might reasonably be accepted as the most plausible because the virus had never been detected before in Brazil. This hypothesis is tied, however, to confirmation by more extensive sampling and phylogenetic analysis.^37^

One important observation in this study is the simultaneous detection of DENV-1 and ZIKV infections in two patients. The *co-infection* of both patients does not appear to cause severe synergistic effects on virulence because both patients had a mild clinical course and made a full recovery without the need for a hospital stay. To our knowledge, only 2 patients with documented DENV and ZIKV coinfection had been reported previously.^39^ The first patient who had detectable DENV-3 and ZIKV had returned from French Polynesia, where ZIKV and DENV were cocirculating.^40,41^ The second case of coinfection by DENV-1 and ZIKV was reported in a patient from New Caledonia who had no travel history. The authors of this study also reported a mild clinical course for both patients, who recovered from the concomitant viral infections without sequel, corroborating with our current report. The evidence of cocirculation and coinfection presented in this study may indicate a high prevalence of the urban vector, which may be capable of transmitting >1 virus at a time.

Historically, infection with ZIKV was documented to cause mild infections in humans. The current outbreak in Brazil, however, witnessed a drastic increase of newborns with congenital microcephaly, as reported by the BMH. Consequently, this raises questions about the possible role of ZIKV infection in congenital microcephaly. Currently, the evidence of an association between the 2 events is ecological and the causative nature of the association cannot be ruled out with the evidence available. Interestingly, our phylogenetic analysis showed that all the 14 Brazilian ZIKV sequences clustered as a distinct group within the Chilean ZIKV sequences, possibly signifying that these viruses had evolved in Brazil. This observation urged us to ask whether the circulating ZIKV viruses in Brazil acquired important genetic changes that make them distinct and more virulent. It is also likely that the emergence of ZIKV in a population already exposed to DENV1-3^42^ and CHIKV, as detected in this study, might be the cause of the increased severity of the disease. In the light of our observations, more studies of ZIKV genomes are needed to provide detailed information on possible changes that might influence ZIKV disease characteristics and vector competence.

Another intriguing point in our study is the fact that most of our patients had small ZIKV virus burdens even in the presence of clinical signs. These results possibly indicate that ZIKV can induce clinical symptoms even at low concentration.

One of the limitations beside the small sample size in our study is the cross-sectional design that permitted obtaining data only at single time points; this limited our ability to investigate the temporality of the virologic burden in relation to disease.

## CONCLUSION

In the present study, we found ZIKV as one of the major emerging arboviruses in this region of Brazil. The viral sequences obtained in this study formed a single cluster that grouped with Chilean and Asiatic lineages. The effect of the cocirculation of DENV, CHIKV, and ZIKV and coinfection of these viruses on severity of the disease needs to be further investigated. Coordinated efforts are necessary to contain the epidemic and continued surveillance is warranted to monitor the incursion and spread of these viruses. Efforts are also necessary to assess the effectiveness of current and future prevention strategies.
